# The empathy and stress mindset of healthcare workers: the chain mediating roles of self-disclosure and social support

**DOI:** 10.3389/fpsyt.2024.1399167

**Published:** 2024-09-12

**Authors:** Jinxia Wu, Jinhua Dou, Daofeng Wang, Lizhuo Wang, Feng Chen, Guohua Lu, Lin Sun, Jianlan Liu

**Affiliations:** ^1^ School of Clinical Medicine, Shandong Second Medical University, Weifang, Shandong, China; ^2^ School of Public Health, Shandong Second Medical University, Weifang, Shandong, China; ^3^ Department of Neurosurgery, Shanting District People’s Hospital, Zaozhuang, Shandong, China; ^4^ School of Practical Teaching Management Department, Shandong Second Medical University, Weifang, Shandong, China; ^5^ School of Psychology, Shandong Second Medical University, Weifang, Shandong, China; ^6^ Management Committee of Shanting Economic Development Zone, Zaozhuang, Shandong, China

**Keywords:** empathy, self-disclosure, social support, stress mindset, healthcare workers

## Abstract

The hospital is a workplace full of stressful events for healthcare workers (HCWs) due to unpredictable changes in their daily routines. Perceptions of stressful events (stress mindset) have a significant impact on an individual’s health and well-being. However, few studies have reported the factors and potential counter mechanisms influencing these perceptions. This study aimed to evaluate the relationship between empathy, self-disclosure, social support, and stress mindset of HCWs, and to explore the mechanism of empathy on stress mindset. Five hundred and eight HCWs (35.2% men and 64.8% women) completed the Interpersonal Reactivity Index (IRI), the Distress Disclosure Index (DDI), the Social Support Rating Scale (SSRS), the Stress Mindset Measure (SMM), and demographic questionnaires online in China. The results showed that empathy was positively linked with stress mindset and positively correlated with self-disclosure and social support. In the multiple mediating model, self-disclosure and social support mediated the association between empathy and stress mindset sequentially. The results imply that empathy, self-disclosure, and social support play a significant role in the formation of HCWs’ stress mindset. These findings have substantial ramifications for reducing stress and creating successful government interventions to fortify stress mindset in healthcare.

## 1 Introduction

### 1.1 Empathy and stress mindset

Due to unpredictable daily occurrences such as medical emergencies at hospitals, healthcare workers (HCWs) frequently encounter stressful situations, compounded by the lack of understanding of their field of work by patients and their families (1), workplace violence (2), and long working hours leading to overwork (3). Stress is described as the tension that develops when an individual believes an event they encounter will create more complications than they can handle, given available resources (4). Stress has a considerable influence on human health (5–7). Research statistics show that, on average, high job stress increases the risk of heart disease by up to 50 percent (8). However, stress also has beneficial effects on the body. New evidence suggests that holding a specific stress mindset has a beneficial effect on people’s health and performance under stress (9, 10).

Crum, Salovey, and Anchor (11) refer to people’s beliefs and impressions of stressful events as the “stress mindset,” which is described as a continuum from “stress is enhancing” to “stress is debilitating.” A high level of stress mindset is representative of a stress-is-enhancing mindset. It is characterized by a positive view of the stressful event and belief that the event will produce a better outcome. This mindset has been shown to boost energy and enhance work performance and life satisfaction (11). The stress -is-debilitating mindset is characterized by a belief that the stressful event will have a negative effect on oneself. Stress mindset has also been linked to increased disease and mortality (12); individuals who believe that “stress is debilitating” are over twice as likely as others to develop coronary heart disease (13). A negative stress mindset can lead to emotional disorders and affect the quality of care from HCWs (14, 15). Given China’s limited healthcare resources and strained doctor–patient interaction, how HCWs view stress is particularly important.

The ability to appropriately experience others’ feelings and comprehend the meaning of those feelings is known as empathy (16). Empathy comprises behavioral (17), cognitive (17), and emotional (18) components, and is directly correlated with one’s personal mindset (19, 20). According to the Russian Doll Model, empathetic individuals utilize various empathy strategies to engage their cognition (e.g., perspective-taking) and emotions (e.g., emotional regulation). These empathy strategies can align with their environment and lead to beneficial outcomes (21). For example, empathy is linked to support for trauma recovery (22), greater emotional resilience (20), and stronger social ties (23). In the context of HCWs, empathy can promote a stress-is-enhancing mindset, and thus reduce burnout and their desire to quit (24–26). Nurses with high levels of empathy not only make patients feel understood and cared for, but higher empathy allows nurses to reconsider their perceptions of stress and find more meaning in their work, which improves their overall psychological state and a stress-is-enhancing mindset (27).

### 1.2 Mediating role of self-disclosure

Self-disclosure is also important in facilitating a stress-is-enhancing mindset in individuals with high levels of empathy (28). Self-disclosure is the sharing of personal information, which serves as a conduit for people to communicate their emotions and thoughts (29). Empathy enhances understanding and trust between people, facilitating deeper self-disclosure. “Compassionate care” and the ability to “think differently” among medical trainees are significantly associated with patient-centered communication variables, which contributes to the establishment of a good doctor–patient relationship and improves communication effectiveness (30). Self-disclosure can be used to reconstruct memories of stressful events. Moreover, it can be utilized to improve our understanding of stressful events through the sharing of inner thoughts with others and by seeing the positive effects of stress, which, in turn, promote a stress-is-enhancing mindset (31). Indeed, sharing stress experiences is linked to wellness (32) and reduced depression and anxiety symptoms (33–35). For instance, Hemenover (36) shows that, after self-disclosure, people with traumatic experiences are able to reduce their psychological stress, transform their self-perceptions, and develop ideas about being more resilient and upwardly mobile in the face of stressful and traumatic events.

### 1.3 Mediating role of social support

Social support describes the financial and emotional support people receive from their social networks, including their families, friends, and organizations (37). Empathy especially contributes to social support (38). Empathy is considered an essential part of a helpful relationship; high levels of empathy are associated with caring behaviors, better interpersonal relationships, and prosocial behaviors (39). It also improves intergroup relations (40), thus facilitating further social support.

According to the social debugging theory of cognitive processing, social support can help individuals change their perceptions of stressful events. Therefore, social support may help people develop a stress-is-enhancing mindset (41). Although this strain of research is at a nascent stage, studies have confirmed the supportive role of social support in generating positive personal results, such as greater enjoyment of life and a sense of hope (42). Individuals with high levels of social support are more likely to develop positive coping mechanisms in the face of stressful events owing to the availability of more social resources; such individuals display a stress-is-enhancing mindset (43).

### 1.4 Chain-mediated effects of self-disclosure and social support

This study also considers whether a relationship exists between the two mediators—self-disclosure and social support. We found that when people talk about their personal experiences, they disclose feelings and information about incidents, which can foster close ties and support (44). Expressing oneself is essential for obtaining social support; social support is unavailable until others are aware of one’s need for assistance. For instance, a study of self-disclosure in timely communication found that, after six months, users who engaged in self-disclosure received higher social support (45). Similarly, male homosexuals who are HIV-positive who self-report more will have more social support exchanges, and thus receive or provide better social support than those who self-report less (46).

### 1.5 Present study

**Figure 1:**
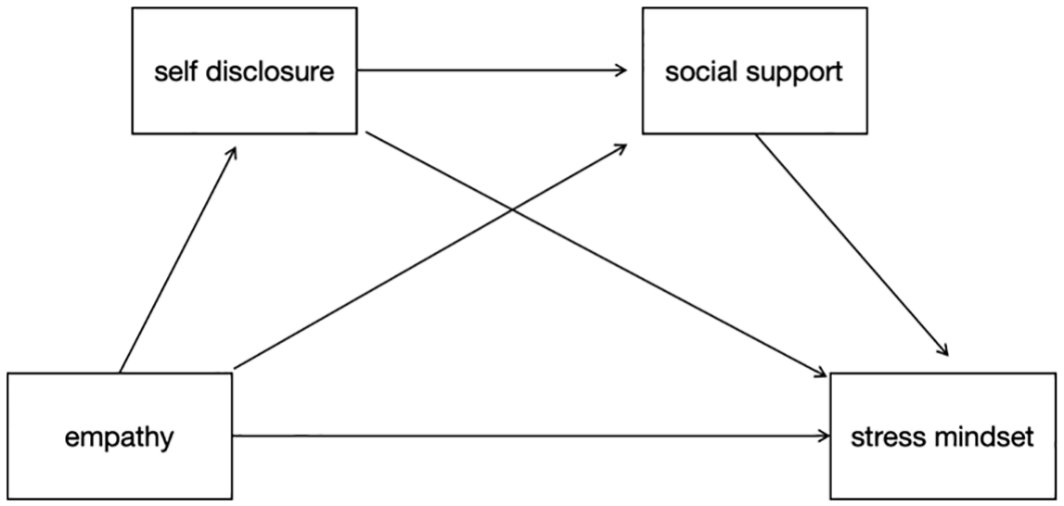
The multiple mediator model of empathy and stress mindset.

While previous research has shown a link between empathy, stress mindset, self-disclosure, and social support, research on the influencing factors of stress mindset in HCWs and the internal mechanisms by which empathy affects stress mindset is still relatively limited. The aim of this study was to investigate the effect of empathy on stress mindset and the mediating role of self-disclosure and social support in HCWs. This study proposes four hypotheses based on these theoretical and empirical foundations (**Figure 1**).

Hypothesis 1. HCWs’ empathy is positively related to their stress mindset.
Hypothesis 2: The association between empathy and stress mindset is mediated by HCWs’ self-disclosure.
Hypothesis 3: The association between empathy and stress mindset is mediated by HCWs’ social support.
Hypothesis 4: Self-disclosure and social support from HCWs mediate the relationship between empathy and stress mindset.

The results of this study will provide a theoretical basis for relevant departments to formulate effective policies and intervention programs to improve the stress mindset of HCWs.

## 2 Methods

### 2.1 Participants

The study was conducted from January to March 2023 and participants were recruited online by WeChat in China. Participants had to meet the following inclusion criteria: (1) be healthcare professionals aged 18 years or older; (2) be able to communicate in standard Chinese to read and understand the questionnaires; and (3) sign the consent form and voluntarily engage in this survey. The Shandong Second Medical University Ethics Committee reviewed and approved the use of human subjects in this study.

We first sent the questionnaire QR code to the leader of the relevant department of a hospital in Zaozhuang, Shandong Province, who then sent it to the WeChat group of healthcare workers. Subsequently, our trained and experienced professionals explained the purpose of this survey to the participants. A total of 622 healthcare professionals participated in the survey, which took approximately 15 minutes to complete. At the end of the questionnaire, we responded to them with a copy of the measurements, an explanation of what they meant, and suggestions or strategies for healthier psyches.

Only 508 of the 622 surveys had valid responses, with an effective recovery rate of 81.67%, of which 179 were male (35.2%) and 329 were female (64.8%) participants (**Table 1**). The respondents completed the Interpersonal Reactivity Index (IRI), the Distress Disclosure Index (DDI), the Social Support Rating Scale (SSRS), the Stress Mindset Measure (SMM), and demographic questionnaires. Each respondent was required to read and comprehend an informed consent form before answering the questionnaire. All participants provided online informed consent to participate in the study.

**Table 1:** Characteristics of participants and stress mindset (N = 508).

Variables	Frequency (percentage)	Stress mindset (M ± SD)	t/F	*p*
Age (years)			0.34	0.887
18–25	9 (1.8)	(17.44 ± 7.06)		
26–30	97 (19.1)	(19.96 ± 5.23)		
31–40	261 (51.4)	(20.49 ± 5.37)		
41–50	98 (19.3)	(20.40 ± 5.08)		
>51	42 (8.3)	(20.57 ± 4.98)		
Sex			0.96	0.327
Male	179 (35.2)	(20.57 ± 5.42)		
Female	329 (64.8)	(20.23 ± 5.28)		
Working years			0.35	0.883
1–5	49 (9.6)	(20.33 ± 5.45)		
6–10	173 (34.1)	(19.95 ± 5.97)		
11–15	132 (26.0)	(20.68 ± 5.04)		
16–20	51 (10.0)	(20.53 ± 5.72)		
21–25	30 (5.9)	(20.30 ± 5.19)		
>26	73 (14.4)	(20.63 ± 5.06)		

### 2.2 Measures

Empathy was measured by the IRI, compiled by Chrysikou (47). The most popular tool for measuring empathy in China is the Chinese version of the Interpersonal Reactivity Index (C-IRI), which was improved by Zhang (48). The internal consistency and reliability Cronbach’s alpha for each factor was 0.60 to 0.77. Twenty-two items were included, and the respondents assessed each item on a 4-point Likert scale (0 = least favorable and 4 = most favorable). This tool has four dimensions: perspective-taking (perspective adjustment), personal pain, empathic care, and fantasy (referring to extended imaging of the events in a situation), with reverse scoring for items 2, 5, 10, 11, and 14. All program scores were added together for a total empathy score. The overall scores ranged from 0 to 88. In our sample, the standardized Cronbach’s alpha value was 0.73, indicating acceptable reliability.

The participants’ self-disclosure was evaluated using the DDI, created by Kahn and Hessling (49). Li of China localized the scale for college students while retaining the original dimensions and entries of the scale (50). The reliability of the revised scale was 0.92, reflecting a high degree of stability. The 12-item index measured distress using a 5-point Likert scale (1 = strong disagreement and 5 = strong agreement). The revised DDI retained the previous 12 items, and items 2, 4, 5, 8, and 10 were reverse-scored entries with a total score ranging from 12 to 60. The sum of the scores for each entry indicated the total self-representation score. Higher scores indicate higher levels of self-disclosure. The standardized Cronbach’s alpha in this study was 0.88, indicating high reliability.

The SSRS was created by Xiao for the Chinese population and includes questions about subjective and objective support as well as their uses and utilization of support (51). The internal consistency and reliability Cronbach’s alpha for each factor was 0.89 to 0.94. In China, the SSRS has been widely applied to numerous populations and has been found to have high reliability and validity. Ten items were included. Items 1, 2, 3, 4, 5, 8, 9, and 10 were scored on a range of 1 to 4 depending on the degree of support (none to some). Items 6 and 7 were scored based on the presence or absence of a support source; the score was determined based on the number of sources, with 0 indicating no support. All program scores were added together for a total social support score. Higher scores indicated more robust social support among the study participants. The total score was the sum of the components. The standardized Cronbach’s alpha in this study was 0.85.

To gauge how people perceive the effects of stress (e.g., “the effects of stress are positive and should be utilized,” “the effects of stress are negative and should be avoided”), Crum et al. created the SMM in 2013 (11). The Chinese He was localized to college students while retaining the original scale (52). The revised scale was more consistent with the localization study, with a Cronbach’s alpha of 0.752–0.828. The scale comprised eight items, and participants were asked to rate each one on a 5-point scale (0 = strongly disagree and 4 = strongly agree). The items in the stress-negative effect dimension were rated in reverse, and then the overall average was determined. The total stress orientation score ranged from 0 to 32. The more points an individual receives, the more likely they are to view difficult situations favorably. The standardized Cronbach’s alpha in this study was 0.80, indicating high reliability.

### 2.3 Data analysis strategy

The common method variance may be present when computerized self-reports are used to acquire data. Consequently, we used techniques during data collection to account for typical method discrepancies, such as screening and reversal questions. The Harman single-factor approach was also applied to all variables obtained from an online self-report method to check for common errors. The findings revealed 19 factors with eigenvalues greater than 1, and the variation described by the first component was 14.25%, falling short of the required value of 40.0%. Thus, the study’s data contained no significant common method variance.

The study used IBM SPSS Statistics for IOS 26.0 for the descriptive statistical analysis. To prepare the data for parametric statistical analysis, the data’s mean, standard deviation, skewness, and kurtosis were examined. The absolute values of skewness and kurtosis did not exceed 1.5 and 3.5, respectively, indicating that the variances of each variable were close to those of a normal distribution (53). In all regression analyses, we entered age, sex, and working years as control variables and conducted structural equation tests for our four hypotheses. Specifically, we examined the structural model using Mplus 8.6 (54).

## 3 Results

### 3.1 Descriptive statistics and correlation analysis

Descriptive statistics, including mean and standard deviation, were calculated for the raw scores of the corresponding questions related to each factor of the study variables.

To better control for measurement error, we utilized raw scores for each topic to estimate the correlation coefficients among the study’s variables. The results confirmed significant positive correlation between empathy, self-disclosure, social support, and stress mindset, two by two (see **Table 2**).

**Table 2:** Descriptive statistics and correlations among variants.

Variable	Empathy	Self-disclosure	Social support	Stress mindset
Empathy	1			
Self-disclosure	0.30**	1		
Social support	0.31**	0.35**	1	
Stress mindset	0.32**	0.31**	0.44**	1
M	67.94	40.74	45.06	20.35
SD	8.32	8.01	7.34	5.33
Skewness	0.46	0.03	1.21	1.12
Kurtosis	1.15	0.16	0.26	2.01

**p<0.01.

### 3.2 Empathy and stress mindset in HCWs: test of chain multiple mediation model

With empathy as the independent variable; stress mindset as the dependent variable; self-disclosure and social support as mediating variables; and gender, age, and working years as controlling variables, a chain mediation model was established. After the examination using Mplus, the model fitting results confirmed model saturation (χ2/df=0, CFI=1.00, TLI=1.00). A Bootstrap method (5000 times) was used to examine the indirect effects of three mediation paths.

The 95% confidence intervals for the indirect effect values of the three mediating paths did not include 0, thereby indicating the significance of the indirect effects across all three paths. This suggests that both the individual mediating roles of self-disclosure and social support, as well as their chained mediating effect, were upheld. Specifically, empathy was strongly and positively related to self-disclosure (β = 0.30, p<0.001), whereas self-disclosure was strongly and positively related to stress mindset (β = 0.09, p<0.01). Further, self-disclosure significantly mediated the relationship between stress mindset and empathy (β = 0.03, p<0.01). As a result, Hypothesis 2 was confirmed. Empathy positively predicted social support (β = 0.17, p<0.001). Social support, in turn, positively predicted stress mindset (β = 0.25, p<0.001). The relationship between empathy and stress mindset was mediated by social support (β = 0.04, p<0.001). Significant social support had a mediating effect. As a result, Hypothesis 3 was confirmed. The mediating effects of self-disclosure and social support on empathy and stress mindset were significant (indirect effect = 0.02, p<0.001). Empathy had a substantial direct relationship with stress mindset (r = 0.12, p<0.001). Consequently, Hypothesis 4 was confirmed (see **Table 3**).

**Table 3:** Direct and indirect effects in the model (Bootstrap method).

	Effect	SE	BootLLCI	BootULCI
TOTAL	0.207	0.034	0.139	0.271
EM → SM	0.116	0.033	0.050	0.180
EM → SD → SM	0.026	0.010	0.009	0.046
EM → SS → SM	0.042	0.010	0.025	0.063
EM → SD → SS → SM	0.024	0.005	0.015	0.035

EM, empathy; SD, self-disclosure; SS, social support; SM, stress mindset.

## 4 Discussion

Given the long-term nature of workplace stress and its effects on mental health (55), examining factors that influence stress mindset may contribute to the well-being of HCWs. However, previous studies have often neglected this topic. The present study examined the connection between empathy and stress mindset among Chinese HCWs to address identified research gaps in the literature.

In line with expectations, self-disclosure and social support simultaneously and sequentially mediated the relationship between empathy and stress mindset among HCWs. These findings advance our knowledge of the relationship between empathy and stress mindset.

Conditional on controlling for influencing variables, we identified the following links between the core variables. We found a positive correlation between empathy and stress mindset among HCWs. Therefore, Hypothesis 1 was supported. Thus, people with higher levels of empathy are more likely to have a stress-is-enhancing mindset. This finding is in line with earlier research; for instance, a study of community workers found a link between higher empathy and higher health mindset levels (15). This result may emerge from the stress incident taking on a new meaning for empathic people whose perceptions have changed. Empathic individuals can think differently and better regulate their emotions through perspective-taking emotional regulation, which allows them to better cope with stress and challenges. They are more likely to transcend the traumatic occurrence, resulting in greater appreciation and a stress-is-enhancing mindset. This conclusion adds to previous studies showing a link between empathy and stress mindset in HCWs.

We also discovered that empathy could affect stress mindset in HCWs through the mediating function of self-disclosure, a finding that lends support to Hypothesis 2. This result aligns with earlier research showing that empathy levels positively correlate with self-disclosure and that self-disclosure is a significant and favorable predictor of stress mindset (56). Empathy positively correlates with self-disclosure in the initial stage of the mediated process (i.e., empathy → self-disclosure). An empathetic person can increase understanding and trust between people and better understand the emotions of others, which improves communication between two people and promotes deeper self-disclosure. However, because of their diminished capacity for empathy, people with low levels of empathy are less likely to indulge in self-disclosure. This finding is consistent with earlier studies that found self-disclosure to be more common among empathic people (56). Self-disclosure and stress mindset positively connect during the second stage of the mediation process. This finding supports the social penetration theory, which contends that self-disclosure is crucial for personal development and the emergence of pleasant emotions. A wealth of credible research supports the link between self-disclosure and stress mindset (57). Through self-disclosure, individuals can obtain feedback and different perspectives from others, which can help them reevaluate and cognitively reframe their perceptions of stressful events. Feedback from others can help individuals recognize the positive side of things or suggest new solutions to change negative perceptions of stress.

Our findings further show that empathy could affect HCWs’ assessment of stress mindset through the mediating function of social support. Thus, Hypothesis 3 was supported. This result confirms earlier studies showing that social support significantly mediates the relationship between empathy and stress mindset (22, 23). According to our findings, those with high levels of empathy obtained higher social support in the initial stage of the mediated process (i.e., empathy → social support). Empathetic individuals tend to put themselves in the other person’s shoes, are more understanding of others; they enjoy closer and stronger relationships and high levels of social support (23). Indeed, we found that greater levels of social support were positively correlated with higher levels of stress mindset for the second component of our mediation model (i.e., social support → stress mindset). This result lends credence to the social penetration hypothesis, which states that people are more likely to undergo personal growth when they have strong social networks. Social support includes objective forms such as material direct assistance as well as subjective forms of personal emotional experience and satisfaction with how they are respected, supported, and understood (58). Good social support contributes to the formation of better interpersonal networks, an improved ability to cope with stress, the avoidance of negative perceptions of stress, and the development of a stress-is-enhancing mindset.

Finally, self-disclosure and social support mediated the relationship between empathy and stress mindset not only in parallel but also in sequence. Therefore, Hypothesis 4 was supported. Individuals with high levels of empathy can cope more effectively with stress and challenges by facilitating stronger social connections and emotional support, which, in turn, creates a stress-is-enhancing mindset. Notably, self-disclosure and social support are positively correlated (44). Further, increased self-disclosure makes people more likely to communicate their needs to others, which increases their chances of receiving assistance and, consequently, their level of social support. However, few studies have combined their analysis of the impact of self-disclosure and social support on stress mindset, despite past research supporting the effects of both factors on stress mindset. By combining the Russian Doll Model theory of empathy and the social penetration hypothesis, we simultaneously accounted for the mediating functions of self-disclosure and social support. Consequently, this integral and paired-chain mediation model offers a more thorough assessment of the connection between empathy and stress mindset.

The findings of the study provide evidence and concrete recommendations for the government and the hospital sector to formulate effective policies and interventions. First, the relevant departments of the hospital should regularly provide psychological counseling to HCWs to help them correctly recognize and deal with empathic emotions and promote transpersonal thinking and emotional regulation. Second, hospitals should create an atmosphere conducive to good communication in order to promote self-disclosure among HCWs, which will further contribute to the formation of their stress-is-enhancing mindset. Finally, when the government formulates policies for HCWs, the content of the assessment and interventions should also pay attention to HCWs’ well-being, since high levels of social support can help promote a stress-is-enhancing mindset.

### 4.1 Limitations

This study has various limitations that should be acknowledged. First, although the mediation model in our study was based on theoretical underpinnings and empirical research, we could not determine causation due to cross-sectional design limitations. Future longitudinal studies are required to validate this model. Second, although the self-report method used in this study can accurately capture participants’ feelings, it invariably produces biased results owing to recall. Finally, the study did not examine additional variables that could affect stress mindset levels, such as active rumination and appreciation. Therefore, further research on the effects of other factors on stress mindset levels is required.

## 5 Conclusions

In summary, this study has significantly advanced the testing of several mediation models related to empathy and stress mindset in a sample of Chinese HCWs. These findings imply that self-disclosure and social support play concurrent and sequential mediating roles in the relationship between empathy and stress mindset. The results of this study will help attenuate stress mindset among HCWs and provide a better understanding of the connection between empathy and stress mindset.

## Data Availability

The raw data supporting the conclusions of this article will be made available by the authors, without undue reservation.
