# Supplementary material for: The empathy and stress mindset of healthcare workers: the chain mediating roles of self-disclosure and social support
Source: Front Psychiatry. 2024 Sep 12;15:1399167. doi: 10.3389/fpsyt.2024.1399167 (PMC11424417; doi:10.3389/fpsyt.2024.1399167)
Supplement: Supplementary file 1 [file DataSheet1.zip › Questionnaire_Chinese.DOCX]

**医护人员心理健康状况调查**

知情同意书
敬爱的医护朋友：
您好！
您现在参加的是一个关于在您所处工作环境下的心理健康状况调查，为对医护朋友进行人文关怀提供科学依据。本研究收集的资料仅学术研究使用，我们将会对您的信息严格保密，请根据您的实际情况如实作答。感谢您的配合。
本次调查可能花费您10～15分钟的时间来完成基本情况和量表的作答。答题时请注意：本问卷的所有答案和结果将完全保密。答案无对错之分，只需您按第一反应回答即可。每一题目都要回答。
可能的受益：
通过本项目的各种测试，您可能对自己的情况有更细致、更全面的了解，这可能有利于您的成长。参加本研究，您还将会得到研究人员提供的相关知识的咨询。
如果您对任何问题有疑问，或需要帮助的话，请联系我们，联系电话：17861202116。
希望和您合作愉快！对您的理解与支持表示万分感谢！
非常荣幸与您相识，并真诚感谢您参与此次调查。
同意参与者签字： [填空题]（提交问卷即为同意）

_________________________________

您的科室是 [单选题]

| ○内科 |
| --- |
| ○外科 |
| ○妇产科 |
| ○儿科 |
| ○精神科 |
| ○其他科室 |

首先，请您回想一下，看看每句话在多大程度上像您。[矩阵量表题]

|  | 完全不符合 | 基本不符合 | 不确定 | 基本符合 | 完全符合 |
| --- | --- | --- | --- | --- | --- |
| 1.我经常对那些没我幸运的人怀有关爱之心。 | ○ | ○ | ○ | ○ | ○ |
| 2.我有时候并不同情那些有困难的人。 | ○ | ○ | ○ | ○ | ○ |
| 3.我真的会陷入小说中人物的情感。 | ○ | ○ | ○ | ○ | ○ |
| 4.我遇到紧急情况时会感到担心和局促不安。 | ○ | ○ | ○ | ○ | ○ |
| 5.我看电影或电视时往往会很客观，也不会完全陷入情节中。 | ○ | ○ | ○ | ○ | ○ |
| 6.我在做决定前会努力考虑每个人的不同意见。 | ○ | ○ | ○ | ○ | ○ |
| 7.我看到某些人被利用时，就想保护他们。 | ○ | ○ | ○ | ○ | ○ |
| 8.我非常激动的时候，有时会感到无助。 | ○ | ○ | ○ | ○ | ○ |
| 9.我有时候会通过想象他人对事情的看法去更好地理解我的朋友。 | ○ | ○ | ○ | ○ | ○ |
| 10.我很少对一本好书或一部好电影极度入迷。 | ○ | ○ | ○ | ○ | ○ |
| 11.我不太会为他人的不幸感到不安。 | ○ | ○ | ○ | ○ | ○ |
| 12.我看完电视或电影时会感到自己就是其中的某个角色。 | ○ | ○ | ○ | ○ | ○ |
| 13.我对紧张的情绪状态感到害怕。 | ○ | ○ | ○ | ○ | ○ |
| 14.我看到某些人受到不公平对待时并不感到他们很可怜。 | ○ | ○ | ○ | ○ | ○ |
| 15.我相信问题都有两面性并会看到问题的两面性。 | ○ | ○ | ○ | ○ | ○ |
| 16.我觉得自己是一个相当富有同情心的人。 | ○ | ○ | ○ | ○ | ○ |
| 17.我看到一部好电影时很容易置身于主角的位置。 | ○ | ○ | ○ | ○ | ○ |
| 18.我在紧急情况时，往往会不知所措。 | ○ | ○ | ○ | ○ | ○ |
| 19.我对某人感到心烦意乱时，通常会设身处地地想想。 | ○ | ○ | ○ | ○ | ○ |
| 20.读到有趣的故事或小说时，我会想如果故事中的事情发生在自己身上的话会有什么感受。 | ○ | ○ | ○ | ○ | ○ |
| 21.我看到别人在紧急情况中孤立无援时，自己会感到身心崩溃。 | ○ | ○ | ○ | ○ | ○ |
| 22.我在批评别人前会努力想假如我是他们，我会有什么感受。 | ○ | ○ | ○ | ○ | ○ |

好了，您已经完成了选择第一部分，接下来，选择第二部分吧～
请您阅读下面的问卷，选出最符合您情况的一项。[矩阵量表题]

|  | 非常不同意 | 比较不同意 | 不确定 | 比较同意 | 非常同意 |
| --- | --- | --- | --- | --- | --- |
| 1.在难过的时候，我通常向朋友倾诉。 | ○ | ○ | ○ | ○ | ○ |
| 2.我不愿谈论自己的问题。 | ○ | ○ | ○ | ○ | ○ |
| 3.当发生不愉快事情时，我经常找人谈论这些事情。 | ○ | ○ | ○ | ○ | ○ |
| 4.我一般不和他人讨论那些使我难过的事情。 | ○ | ○ | ○ | ○ | ○ |
| 5.当感到沮丧或难过时，我总是独自承担。 | ○ | ○ | ○ | ○ | ○ |
| 6.我会找人谈论自己的问题。 | ○ | ○ | ○ | ○ | ○ |
| 7.当心情不好时，我会找朋友聊天。 | ○ | ○ | ○ | ○ | ○ |
| 8.如果难过，我最不愿意找别人倾诉。 | ○ | ○ | ○ | ○ | ○ |
| 9.当遇到难处时，我很少找别人谈论这些困难。 | ○ | ○ | ○ | ○ | ○ |
| 10.当痛苦的时候，我不会告诉任何人。 | ○ | ○ | ○ | ○ | ○ |
| 11.当心情不好的时候，我一般找人聊天。 | ○ | ○ | ○ | ○ | ○ |
| 12.我愿意把不高兴的事告诉别人。 | ○ | ○ | ○ | ○ | ○ |

下面这个题请您选a选项 [单选题]

| ○a |
| --- |
| ○b |
| ○c |
| ○d |

您对压力的看法是什么？
请您阅读下面的问卷，选择最符合您想法的一项。[矩阵量表题]

|  | 非常不同意 | 不同意 | 不确定 | 同意 | 非常同意 |
| --- | --- | --- | --- | --- | --- |
| 1.压力的影响是负面的，应该避免。 | ○ | ○ | ○ | ○ | ○ |
| 2.经历压力有助于我的学习和成长。 | ○ | ○ | ○ | ○ | ○ |
| 3.经历压力会耗尽我的健康和活力。 | ○ | ○ | ○ | ○ | ○ |
| 4.经历压力可以提高我的表现和工作效率。 | ○ | ○ | ○ | ○ | ○ |
| 5.经历压力会抑制我的学习和成长。 | ○ | ○ | ○ | ○ | ○ |
| 6.经历压力可以改善我的健康和活力。 | ○ | ○ | ○ | ○ | ○ |
| 7.经历压力会削弱我的表现和工作效率。 | ○ | ○ | ○ | ○ | ○ |
| 8.压力的影响是积极的，应该加以利用。 | ○ | ○ | ○ | ○ | ○ |

请按各个问题的具体要求，根据您的实际情况填写。
1. 您有多少关系密切，可以得到支持和帮助的朋友？（只选一项） [单选题]

| ○（1）1个也没有 |
| --- |
| ○（2）1－2个 |
| ○（3）3－5个 |
| ○（4）6个或 6个以上 |

2. 近一年来您：（只选一项） [单选题]

| ○（1）远离家人，且独居一室 |
| --- |
| ○（2）住处经常变动，多数时间和陌生人住在一起 |
| ○（3）和同学、同事或朋友住在一起 |
| ○（4）和家人住在一起 |

3. 您和邻居：（只选一项） [单选题]

| ○（1）相互之间从不关心，只是点头之交 |
| --- |
| ○（2）遇到困难可能稍微关心 |
| ○（3）有些邻居很关心您 |
| ○（4）大多数邻居都很关心您 |

4. 您和同事：（只选一项） [单选题]

| ○（1）相互之间从不关心，只是点头之交 |
| --- |
| ○（2）遇到困难可能稍微关心 |
| ○（3）有些同事很关心您 |
| ○（4）大多数同事都很关心您 |

5. 以下是您从家庭成员得到的支持和照顾，请选出最符合您情况的一项[矩阵量表题]

|  | 无 | 极少 | 一般 | 全力支持 |
| --- | --- | --- | --- | --- |
| A、夫妻（恋人） | ○ | ○ | ○ | ○ |
| B、父母 | ○ | ○ | ○ | ○ |
| C、儿女 | ○ | ○ | ○ | ○ |
| D、兄弟姐妹 | ○ | ○ | ○ | ○ |
| E、其他成员 | ○ | ○ | ○ | ○ |

6. 过去，在您遇到急难情况时，曾经得到的经济支持和解决实际问题的帮助的来源有： [单选题]

| ○无任何来源 (请跳至第7题) |
| --- |
| ○下列来源（可选多项） |

来源选择 [多选题]

| □A、配偶 |
| --- |
| □B、其他家人 |
| □C、朋友 |
| □D、亲戚 |
| □E、同事 |
| □F、工作单位 |
| □G、党团工会等官方或半官方组织 |
| □H、宗教、社会团体等非官方组织 |
| □I、其它 |

依赖于第6.题第2个选项

7. 过去，在您遇到急难情况时，曾经得到的安慰和关心的来源有： [单选题]

| ○无任何来源 (请跳至第8题) |
| --- |
| ○下列来源（可选多项） |

来源选择 [多选题]

| □A、配偶 |
| --- |
| □B、其他家人 |
| □C、朋友 |
| □D、亲戚 |
| □E、同事 |
| □F、工作单位 |
| □G、党团工会等官方或半官方组织 |
| □H、宗教、社会团体等非官方组织 |
| □I、其它 |

依赖于第7.题第2个选项

8. 您遇到烦恼时的倾诉方式：（只选一项） [单选题]

| ○（1）从不向任何人诉说 |
| --- |
| ○（2）只向关系极为密切的 1－2 个人诉说 |
| ○（3）如果朋友主动询问您会说出来 |
| ○（4）主动诉说自己的烦恼以获得支持理解 |

9. 您遇到烦恼时的求助方式：（只选一项） [单选题]

| ○（1）只靠自己，不接受别人帮助 |
| --- |
| ○（2）很少请求别人帮助 |
| ○（3）有时请求别人帮助 |
| ○（4）有困难时经常向家人、亲友、组织求援 |

10. 对于团体（如党组织、宗教组织、工会、学生会等）组织的活动，您：（只选一项） [单选题]

| ○（1）从不参加 |
| --- |
| ○（2）偶尔参加 |
| ○（3）经常参加 |
| ○（4）主动参加并积极活动 |

基本资料调查：请填写您的个人资料

您的性别： [单选题]

| ○男 | ○女 |  |  |  |  |  |  |
| --- | --- | --- | --- | --- | --- | --- | --- |

婚姻状况： [单选题]

| ○已婚 |
| --- |
| ○未婚 |
| ○离异或丧偶 |

业务职称： [单选题]

| ○初级 |
| --- |
| ○中级 |
| ○副高级 |
| ○正高级 |

年龄： [单选题]

| ○18岁以下 |
| --- |
| ○18~25 |
| ○26~30 |
| ○31~40 |
| ○41~50 |
| ○51~60 |
| ○60以上 |

工作年限： [单选题]

| ○1~5 |
| --- |
| ○6~10 |
| ○11~15 |
| ○16~20 |
| ○21~25 |
| ○26以上 |

教育程度 [单选题]

| ○初中 |
| --- |
| ○高中 |
| ○大学本科 |
| ○硕士研究生 |
| ○博士研究生 |

联系方式（用于测评结果反馈） [填空题]

_________________________________

调查到此结束了，感谢您的参与！未来工作的日子里也要加油哦～祝您生活愉快，工作顺利！期待和您下次相见，如果您想获得此次结果的反馈，您可以留下您的电话：我们届时将会以短信的形式将结果发送给您。您也可以拨打我们的电话（微信），17861202116；邮箱：17861202116@163.com 询问结果再次表示对您的感谢！再见！
