# Supplementary material for: The empathy and stress mindset of healthcare workers: the chain mediating roles of self-disclosure and social support
Source: Front Psychiatry. 2024 Sep 12;15:1399167. doi: 10.3389/fpsyt.2024.1399167 (PMC11424417; doi:10.3389/fpsyt.2024.1399167)
Supplement: Supplementary file 1 [file DataSheet1.zip › Questionnaire_English.PDF]

yd 有道翻译 支持免费文档翻译240万字符/年 立即下载→ https://fanyi.youdao.com/download

Investigation of mental health status of medical staff

Informed Consent

Dear medical friends:

Hello!

You are now participating in a mental health survey in your working environment, which provides scientific basis for humanistic care for medical friends. The data collected in this study is for academic research only, and we will keep your information strictly confidential. Please answer truthfully according to your actual situation. Thank you for your cooperation. This survey may take you 10 to 15 minutes to complete the basic information and the answers to the scale. Please note: All answers and results of this questionnaire will be kept completely confidential. There are no right or wrong answers. You just need to answer as your first response. You should answer every question.

Possible benefits:

Through the various tests of this program, you may have a more nuanced and comprehensive understanding of your condition, which may benefit your growth. By participating in the study, you will also be consulted by the researchers about the relevant knowledge.

If you have any questions or need help, please contact us at 17861202116.

Hope to cooperate with you happily! Thank you very much for your understanding and support.

It is a great honor to make your acquaintance, and I sincerely thank you for participating in this survey.

Consent Participant Signature: [Fill in the blanks] (Submission of the questionnaire is consent)

____

Your department is [multiple choice]

Internal Medicine

○ Surgery

○ Obstetrics and Gynecology

○ Pediatrics

○ Psychiatry

○ Other Departments

First, I ask you to think back and see to what extent each sentence is like you. [Matrix Scale question]

	Completely inconsistent	Not at all	Inconclusive	Basically match	A perfect match
1. I always have compassion for those less fortunate than myself.	a.	a.	a.	a.	a.
2. Sometimes I don't sympathize with people who are in trouble.	a.	a.	a.	a.	a.

3. I really get into the emotions of the characters in the novel.	a.	a.	a.	a.	a.
4. I feel worried and embarrassed when I have an emergency.	a.	a.	a.	a.	a.
5. When I watch movies or TV, I tend to be objective and not completely caught up in the plot.	a.	a.	a.	a.	a.
I try to consider	a.	a.	a.	a.	a.

everyone's different opinions before I make a decision.					
7. When I see someone being used, I want to protect them.	a.	a.	a.	a.	a.
8. When I'm very excited, I sometimes feel helpless.	a.	a.	a.	a.	a.
9. I sometimes try to understan d my friends better by imagining how they	a.	a.	a.	a.	a.

see things.					
10. I rarely get too caught up in a good book or movie.	a.	a.	a.	a.	a.
11. I'm not very upset by the misfortunes of others.	a.	a.	a.	a.	a.
12. When I finish watching TV or a movie, I feel like I'm a character.	a.	a.	a.	a.	a.
13. I am afraid of intense emotional states.	a.	a.	a.	a.	a.
14. I don't feel sorry for	a.	a.	a.	a.	a.

someone when I see them being treated unfairly.					
I believe there are two sides to every problem and I see both sides.	a.	a.	a.	a.	a.
16. I consider myself a fairly compassionate person.	a.	a.	a.	a.	a.
When I see a good movie, it's easy for me to be in the leading role.	a.	a.	a.	a.	a.
18. I tend	a.	a.	a.	a.	a.

to be at a loss in emergency situations.					
19. When I'm upset about someone, I usually try to put myself in their shoes.	a.	a.	a.	a.	a.
20. When I read an interesting story or novel, I think about how I would feel if the things in the story happened to me.	a.	a.	a.	a.	a.
When I see others	a.	a.	a.	a.	a.

alone in an emergency, I feel physically and mentally broken.					
22. I try to think about how I would feel in their shoes before I criticize others.	a.	a.	a.	a.	a.

Ok, you have finished choosing Part 1. Now, choose Part 2.

Please read the questionnaire below and choose the one that best suits your situation. [Matrix Scale Question]

	Strongly disagree	Somewhat disagree	Not sure	More in agreement	Strongly agree
1. When I am sad, I usually confide in my friends.	a.	a.	a.	a.	a.

2. I don't like to talk about my problems.	a.	a.	a.	a.	a.
3. When unpleasant things happen, I often find someone to talk to about them.	a.	a.	a.	a.	a.
4. I don't discuss things that upset me with others.	a.	a.	a.	a.	a.
5. When I feel down or sad, I always take it on myself.	a.	a.	a.	a.	a.
6. I talk to someone about my problems.	a.	a.	a.	a.	a.

7. When I am in a bad mood, I talk to my friends.	a.	a.	a.	a.	a.
8. If I'm sad, the last thing I want to do is talk to someone else.	a.	a.	a.	a.	a.
9. When I encounter difficulties, I seldom turn to others to talk about them.	a.	a.	a.	a.	a.
10. When I'm in pain, I don't tell anyone.	a.	a.	a.	a.	a.
11. When I am in a bad mood,	a.	a.	a.	a.	a.

I usually talk to someone.					
12. I am willing to tell others when I am unhappy.	a.	a.	a.	a.	a.

Please choose a (multiple choice) for the following question.

○a

○b

○c

○d

How do you feel about stress?

Please read the following questionnaire and choose the one that best matches your opinion. [Matrix Scale Question]

	Strongly disagree	Disagree	Not sure	agree.	Couldn't agree more
1. The effects of stress are negative and should be avoided.	a.	a.	a.	a.	a.

2. Experiencing stress helps me learn and grow.	a.	a.	a.	a.	a.
3. Experiencing stress drains my health and vitality.	a.	a.	a.	a.	a.
4. Experiencing stress improves my performance and productivity.	a.	a.	a.	a.	a.
5. Experiencing stress inhibits my learning and growth.	a.	a.	a.	a.	a.

6. Experiencing stress improves my health and vitality.	a.	a.	a.	a.	a.
7. Experiencing stress impairs my performance and productivity.	a.	a.	a.	a.	a.
8. The effects of stress are positive and should be harnessed.	a.	a.	a.	a.	a.

Please fill in according to the specific requirements of each question and your actual situation.

1. How many close friends do you have that you can count on for support and help? (Choose one item only) [Multiple choice]

○ (1) None

○ (2) 1-2

○ (3) 3-5

○ (4) 6 or more

2. In the past year you: (Choose only one item) [Multiple choice]

○ (1) You live in a single room away from your family

○ (2) They move around a lot and live with strangers most of the time

○ (3) Living with classmates, colleagues or friends

○ (4) Living with family

3. You and your neighbors: (Choose one) [multiple choice]

○ (1) You and your neighbor never care about each other. They are just nodding acquaintances

○ (2) We may show little concern when we are in trouble

○ (3) Some neighbors are very concerned about you

○ (4) Most of your neighbors care about you

4. You and your colleagues: (Choose one item only) [Multiple choice]

○ (1) You don't care about each other. You just have a nodding acquaintance

○ (2) We may show little concern when we are in trouble

○ (3) Some colleagues care about you

○ (4) Most colleagues care about you

5. Below are the types of support and care you receive from your family members. Please choose the one that best suits your situation.

	There is no	Very few	Average	Full support

A. Husband and wife (lovers)	a.	a.	a.	a.
B) Parents	a.	a.	a.	a.
C. Children	a.	a.	a.	a.
D) Siblings	a.	a.	a.	a.
E, other members	a.	a.	a.	a.

6. In the past, when you were in an emergency situation, the sources of financial support and practical help you have received are: [multiple choice]

○ No source (Please skip to question 7)

○ The following sources (multiple options)

Source selection [multiple choice]

□ A. Spouse

□B. Other family members

□C. Friends

□D, relatives

□E, colleagues

□F, work unit

□G. Official or semi-official organizations such as party and group trade unions

□H, religious, social groups and other non-official organizations

□I, other

Depends on option 2 of question 6

7. In the past, when you met with emergency situations, the sources of comfort and concern you used to get are: [multiple choice]

○ No source (Please skip to question 8)

○ The following sources (multiple options)

Source selection [multiple choice]

□ A. Spouse

□B. Other family members

□C. Friends

□D, relatives

□E, colleagues

□F, work unit

□G. Official or semi-official organizations such as party and group trade unions

□H, religious, social groups and other non-official organizations

□I, other

Depends on option 2 of question 7

8. The way you talk about your troubles: (Choose only one) [multiple choice]

○ (1) You never tell anyone

○ (2) Only to one or two very close people

○ (3) You will tell your friends if they ask

○ (4) Take the initiative to talk about your troubles to get support and understanding

9. How to help you when you are troubled: (Choose only one) [Multiple choice]

○ (1) Rely only on yourself and do not accept help from others

○ (2) Rarely asks for help

○ (3) Sometimes ask others for help

○ (4) Often ask for help from family, relatives and friends, and organizations when in trouble

10. For activities organized by groups (such as party organizations, religious organizations, trade unions, student unions, etc.), you: (choose only one) [single choice]

○ (1) Never participate

○ (2) Occasionally

○ (3) Attend regularly

○ (4) Take the initiative to participate and be active

Basic information survey: Please fill in your personal information

Your gender: [multiple choice]

○ Male ○ Female

Marital status: [multiple choice]

Married -

○ Unmarried

○ Divorced or widowed

Professional title: [multiple choice]

○ Junior

○ Intermediate

○ Associate Advanced

○ Senior

Age: [multiple choice]

○ Age 18 or younger

18 ~ 25

26 ~ 30

31 to 40

41 to 50

51 to 60

○60 +

Years of service: [multiple choice]

1 ~ 5

6 ~ 10

11 ~ 15

16 ~ 20

21 to 25

○26 +

Education [multiple choice]

Junior High School -

○ High School

College undergraduate

○ Graduate Students

○ Doctoral Candidates

Contact information (for assessment results feedback) [Fill in the blanks]

____

That concludes the survey, thanks for your participation! I wish you a happy life and smooth work in the future. Look forward to seeing you next time. If you would like to get feedback on this result, you can leave your phone number: we will send you the result by SMS. You can also call us on wechat at 17861202116; Email: 17861202116@163.com We would like to express our thanks to you again for asking the result! Bye!
